# Supplementary material for: Direct production of low-oxygen-concentration titanium from molten titanium
Source: Nat Commun. 2024 Jun 12;15:5015. doi: 10.1038/s41467-024-49085-4 (PMC11169373; doi:10.1038/s41467-024-49085-4)
Supplement: Supplementary file 1 — Supplementary Information [file 41467_2024_49085_MOESM1_ESM.pdf]

Supplementary Information

Direct Production of Low-Oxygen-Concentration Titanium from Molten Titanium

Toru H. Okabe[1,*], Gen Kamimura[1], Takashi Ikeda[1] & Takanari Ouchi[1,*]

[1]Institute of Industrial Science, The University of Tokyo, Tokyo, Japan.

*Corresponding authors:

Toru H. Okabe: okabe@iis.u-tokyo.ac.jp

Takanari Ouchi: t-ouchi@iis.u-tokyo.ac.jp

This file includes:

Supplementary Figures 1–6

Supplementary Tables 1–5

Supplementary References

Details on the thermodynamic evaluation on deoxidation of liquid Ti

Supplementary Table 1

Thermodynamic data of the compounds in the Y–O–F systems and oxygen dissolution into liquid Ti at 2000 K (1727 °C)

Reaction	Standard Gibbs energy change of reaction at 2000 K (1727 °C), ΔG°_r / J·mol^{-1}	Supplementary Ref. no.
2 Y (*l*) + 3/2 O_2 (*g*) = Y_2O_3 (*s*)	− 1,333,000	1
Y (*l*) + 3/2 F_2 (*g*) = YF_3 (*l*)	− 1,266,000	1
Y (*l*) + 1/2 O_2 (g) + 1/2 F_2 (*g*) = YOF (*s*)	− 907,400	1–3
1/2 O_2 (*g*) = O (1 mass% in Ti (*l*))	− 400,000 [a]	4–7

[a] Rough estimation based on the literature values.

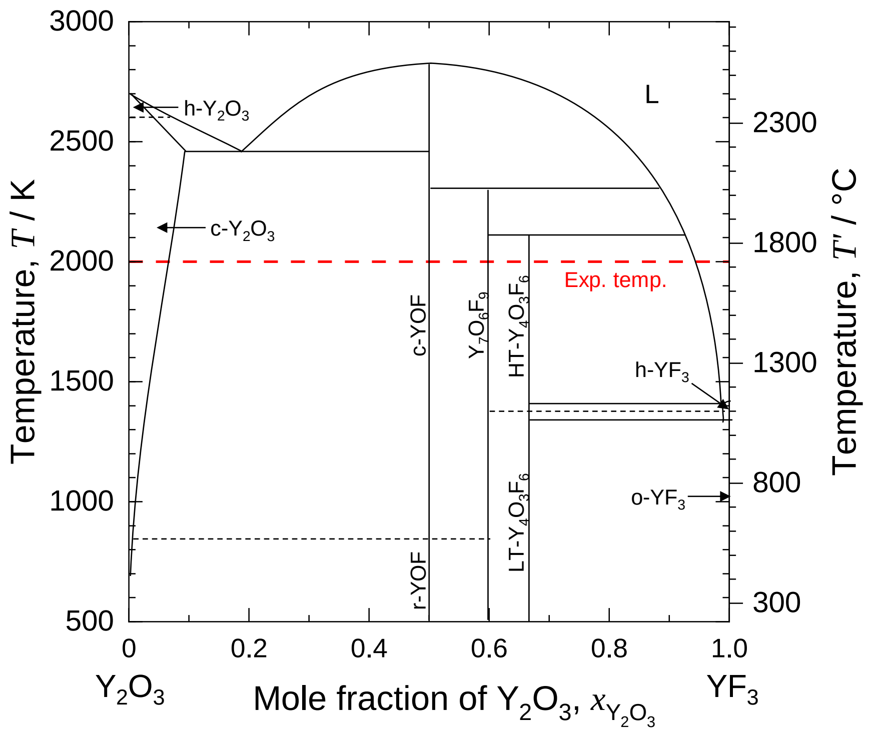

Supplementary Figure 1

Phase diagram for the Y_2O_3–YF_3 pseudobinary system[2]. Based on this diagram, liquid YF_3 phase can equilibrate with the yttrium oxyfluoride phases even at high temperatures above the melting point of Ti.

Details on the analysis of oxygen and nitrogen concentrations in metal samples

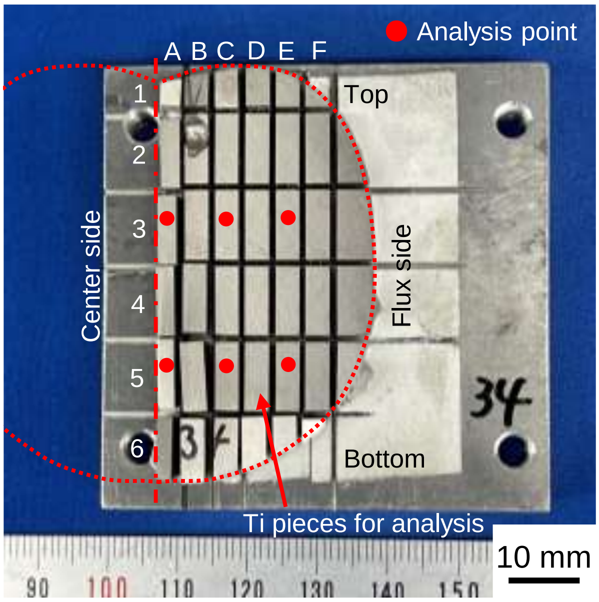

Supplementary Figure 2

Analysis positions of the Ti sample in Exp. #1. The Ti sample was analyzed for oxygen and nitrogen concentrations by the inert gas fusion method (LECO ON836). As shown in Supplementary Table 2, the deoxidation reaction proceeded uniformly in the entire sample. The red dotted line surrounds the Ti sample after deoxidation, which was cut on a base plate and divided into small pieces.

Supplementary Table 2

The oxygen and nitrogen concentrations of the Ti samples in Exp. #1

Exp. #	Analysis Position [a]	Oxygen conc., C_O (mass ppm) Initial (nominal), $C_{O,initial}$ [b]	After, $C_{O,after}$ [c1]	Analysis error, $\varepsilon_{O,after}$ (%)	Nitrogen conc., C_N (mass ppm) Initial (nominal), $C_{N,initial}$ [b]	After, $C_{N,after}$ [c2]	Analysis error, $\varepsilon_{N,after}$ (%)
1	A3	~1000	170	6	~30	140	4
	A3		220	5		140	4
	A5		110	8		130	4
	A5		120	8		120	4
	C3		140	7		130	4
	C3		230	5		150	4
	C5		110	8		140	4
	C5		120	8		120	4
	E3		190	6		140	4
	E5		120	8		150	4

[a] See Supplementary Figure 2.

[b] The initial concentrations of oxygen and nitrogen in the Ti sample were calculated from the weight and composition of the input Ti materials.

Analysis conditions:

[c1] Blank: graphite crucible, part number 782-720, 1 g, 0.0 ± 0.1 μg O, 0.0 ± 0.1 μg N;
Flux: Ni basket, part number 502-344, 1 g, 1.3 ± 0.7 μg O, 0.3 ± 0.2 μg N;
Standard sample for calibration: steel pin, part number 502-874, 1 g, 366 ± 6 ppm O, 23 ± 2 ppm N.

[c2] Blank: graphite crucible, part number 782-720, 1 g, 0.0 ± 0.1 μg O, 0.0 ± 0.1 μg N;
Flux: Ni basket, part number 502-344, 1 g, 1.4 ± 0.7 μg O, 0.3 ± 0.2 μg N;
Standard sample for calibration: steel pin, part number YY-001-125, 1 g, 89 ± 8 ppm O, 432 ± 9 ppm N.

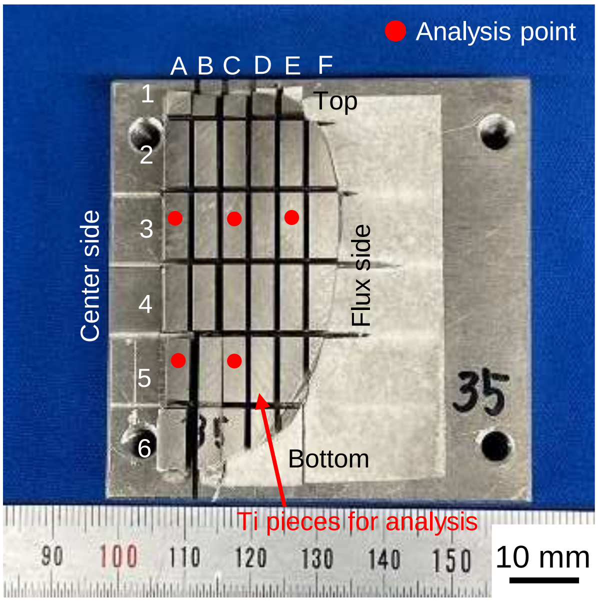

Supplementary Figure 3
Analysis positions of the Ti sample in Exp. #2. The Ti sample was analyzed for oxygen and nitrogen concentrations by the inert gas fusion method (LECO ON836). As shown in Supplementary Table 3, the deoxidation reaction proceeded uniformly in the entire sample. Ti pieces were prepared as in Supplementary Figure 2.

Supplementary Table 3
The oxygen and nitrogen concentrations of the Ti samples in Exp. #2.

		Oxygen conc., C_O (mass ppm)			Nitrogen conc., C_N (mass ppm)		
Exp. #	Analysis position [a]	Initial (nominal), $C_{O,initial}$ [b]	After, $C_{O,after}$	Analysis error, $\varepsilon_{O,after}$ (%)	Initial (nominal), $C_{N,initial}$ [b]	After, $C_{N,after}$	Analysis error, $\varepsilon_{N,after}$ (%)
2	A3	~1000	600 [c1]	3	~30	100 [c2]	4
	A5		580 [c1]	3		100 [c2]	4
	C3		620 [c1]	3		100 [c2]	4
	C5		580 [c1]	3		70 [c2]	5
	E3		600 [c1]	3		110 [c2]	4

[a] See Supplementary Figure 3.
[b] The initial concentrations of oxygen and nitrogen in the Ti sample were calculated from the weight and composition of the input Ti materials.
Analysis conditions:
[c1] Blank: graphite crucible, part number 782-720, 1 g, 0.0 ± 0.1 μg O, 0.0 ± 0.1 μg N;
Flux: Ni basket, part number 502-344, 1 g, 1.3 ± 0.7 μg O, 0.3 ± 0.2 μg N;
Standard sample for calibration: steel pin, part number 502-874, 1 g, 366 ± 6 ppm O, 23 ± 2 ppm N.
[c2] Blank: graphite crucible, part number 782-720, 1 g, 0.0 ± 0.1 μg O, 0.0 ± 0.1 μg N;
Flux: Ni basket, part number 502-344, 1 g, 1.4 ± 0.7 μg O, 0.3 ± 0.2 μg N;
Standard sample for calibration: steel pin, part number YY-001-125, 1 g, 89 ± 8 ppm O, 432 ± 9 ppm N.

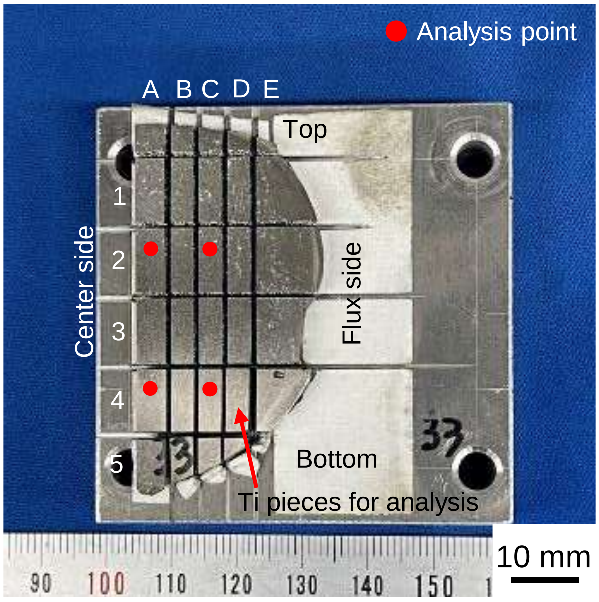

Supplementary Figure 4

Analysis positions of the Ti sample in Exp. #8. The Ti sample was analyzed for oxygen and nitrogen concentrations by the inert gas fusion method (LECO ON836). As shown in Supplementary Table 4, the deoxidation reaction proceeded uniformly in the entire sample. Ti pieces were prepared as in Supplementary Figure 2.

Supplementary Table 4

The oxygen and nitrogen concentrations of the Ti samples in Exp. #8.

		Oxygen conc., C_O (mass ppm)			Nitrogen conc., C_N (mass ppm)		
Exp. #	Analysis position [a]	Initial (nominal), $C_{O,initial}$ [b]	After, $C_{O,after}$	Analysis error, $\varepsilon_{O,after}$ (%)	Initial (nominal), $C_{N,initial}$ [b]	After, $C_{N,after}$	Analysis error, $\varepsilon_{N,after}$ (%)
8	A2	~10000	1100 [c1]	3	~30	240 [c2]	3
	A4		860 [c1]	3		220 [c2]	3
	C2		860 [c1]	3		230 [c2]	3
	C4		780 [c1]	3		230 [c2]	3

[a] See Supplementary Figure 4.

[b] The initial concentrations of oxygen and nitrogen in the Ti sample were calculated from the weight and composition of the input Ti materials.

Analysis conditions:

[c1] Blank: graphite crucible, part number 782-720, 1 g, 0.0 ± 0.1 μg O, 0.0 ± 0.1 μg N;
Flux: Ni basket, part number 502-344, 1 g, 1.3 ± 0.7 μg O, 0.3 ± 0.2 μg N;
Standard sample for calibration: steel pin, part number 502-874, 1 g, 366 ± 6 ppm O, 23 ± 2 ppm N.

[c2] Blank: graphite crucible, part number 782-720, 1 g, 0.0 ± 0.1 μg O, 0.0 ± 0.1 μg N;
Flux: Ni basket, part number 502-344, 1 g, 1.4 ± 0.7 μg O, 0.3 ± 0.2 μg N;
Standard sample for calibration: steel pin, part number YY-001-125, 1 g, 89 ± 8 ppm O, 432 ± 9 ppm N.

Details on the analysis of yttrium oxyfluoride phases in fluxes

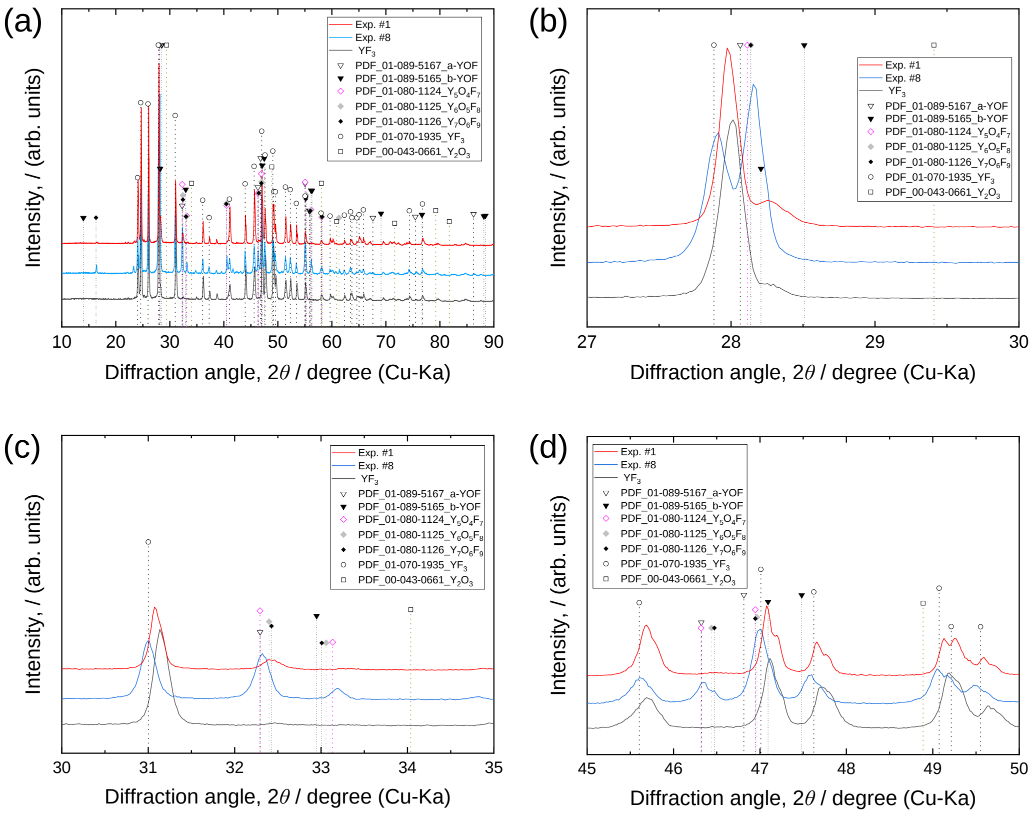

Supplementary Figure 5

XRD patterns of fluxes after the experiments and initial YF_3 reagent. Diffraction angles (2*θ*): (a) 10–90 degrees, (b) 27–30 degrees, (c) 30–35 degrees, and (d) 45–50 degrees. The YF_3 peaks seemed to have shifted to the higher angle side. In the Exp. #8, a new large peak was observed at high angle near 28 degrees. It was considered to be a peak of a compound consisting of Y–O–F. Peaks around 32.5 degrees and 33 degrees in the fluxes of Exps. #1 and #8 were thought to be due to a compound composed of Y–O–F. There was a peak around 46.5 degree in the flux of Exp. #8, which may be attributed to a compound composed of Y–O–F. Y_2O_3 peaks were not observed at 29.4 degrees, 34 degrees, and 48.9 degrees.

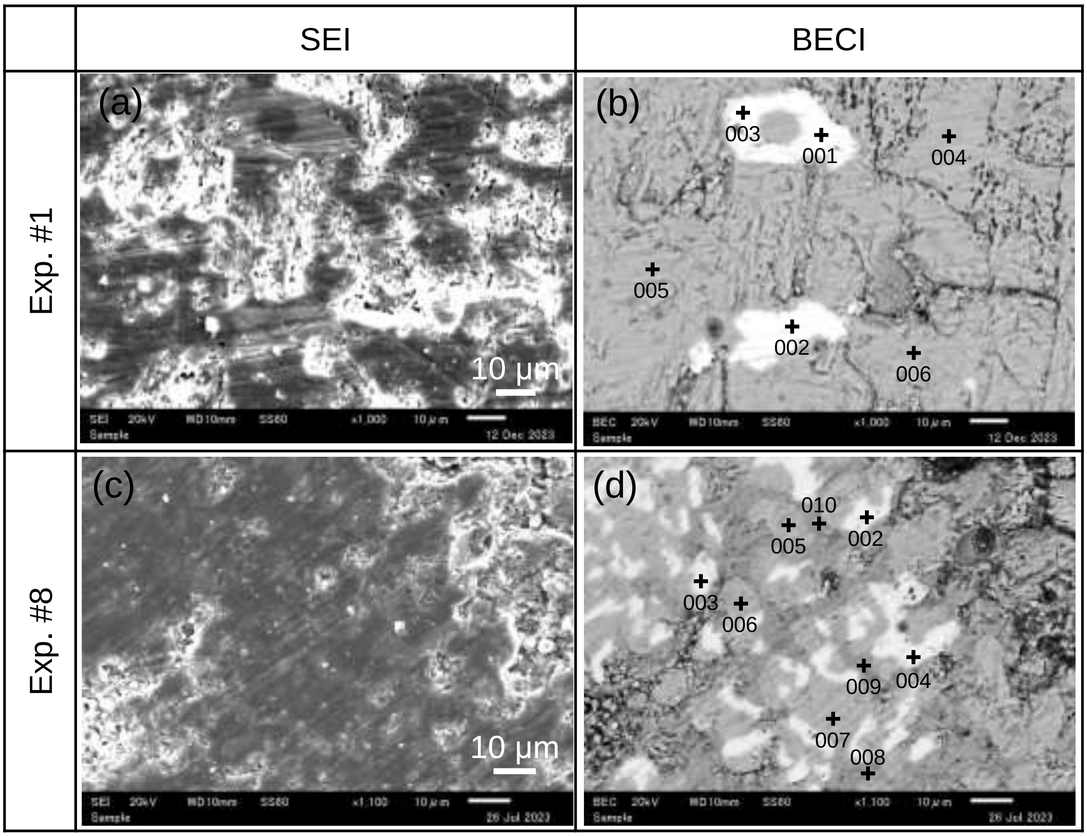

Supplementary Figure 6

SEM images (secondary electron images (SEIs) and backscattered electron composition images (BECIs)) of fluxes after the experiments: (a) SEI and (b) BECI for Exp. #1; (c) SEI and (d) BECI for Exp. #8. EDS results for markers in the BECIs are shown in Supplementary Table 5. According to the BECI of the flux in Exp. #8, there were white regions for Y metal, light gray regions for Y–O–F compounds, and dark gray regions for YF_3. This result indicates that the $Y/YOF/YF_3$ equilibrium was achieved in the current systems. For the flux in Exp. #1, the existence of Y–O–F compound was not clearly observed. This may be due to the results that the amount of compounds produced was small during the deoxidation.

Supplementary Table 5

EDS analysis results of the fluxes in Exps. #1 and #8 (cf. Supplementary Figure 6)

Exp. #	Analysis point *	Concentration of element *i*, C_i (mol%)			Phase
		Y	O	F	
1	001	86.7	10.7	2.6	Metallic Y
	002	89.0	8.5	2.5	
	003	86.0	11.1	2.8	
	004	41.4	2.8	55.8	YF_3 **
	005	42.0	2.4	55.6	
	006	43.2	2.1	54.7	
8	002	88.2	9.9	1.9	Metallic Y
	003	84.8	10.3	4.9	
	004	85.9	11.6	2.5	
	005	48.2	19.6	32.3	Yttrium oxyfluoride
	006	48.1	19.0	32.9	
	007	48.9	21.5	29.6	
	008	38.6	5.8	55.5	YF_3 **
	009	43.9	7.3	48.8	
	010	41.3	5.3	53.4	

* The analysis points are shown in Supplementary Figure 5.

** The Y concentration of initial YF_3 reagent was 37–42 mol% based on the EDS analysis.

Supplementary References

1. Barin, I. *Thermochemical data of pure substances, 3rd ed.* (Wiley-VCH, 1995).
2. Baek, S. & Jung, I.-H. Phase diagram study and thermodynamic assessment of the Y_2O_3-YF_3 system. *J. Eur. Ceram. Soc.* **42**, 5079–5092 (2022).
3. Levitskii, V. A. & Balak, G. M. Determination of the thermodynamic properties of the oxide fluorides of yttrium and of the rare-earth metals by the e.m.f. method with a fluoride ion electrolyte. *Russ. J. Phys. Chem.* **56**, 668–673 (1982).
4. Chang, Y. A. & Hu, D. C. On the gibbs energy interaction parameters of oxygen and nitrogen in liquid alloys. *Metall. Trans. B* **10**, 43–48 (1979).
5. Liang, W. W. & Schuster, W. Solubility of Oxides in Liquid Alloys Containing Titanium and Zirconium. *Trans. Jpn. Inst. Met.* **23**, 368–377 (1982).
6. Maeda, M., Yahata, T. & Ikeda, T. Aluminothermic Reduction of Titanium Oxide. *Mater. Trans., JIM* **34**, 599–603 (1993).
7. Kobayashi, Y. & Tsukihashi, F. Thermodynamics of Oxygen in Molten Ti-Al and Zr-Al Alloys. *High Temp. Mater. Processes* **19**, 211–218 (2000).
